# Extreme heat impacts on daily life and adaptive behaviours captured through lived experience

**DOI:** 10.1088/1748-9326/adcbc5

**Published:** 2025-05-06

**Authors:** Joanne L Godwin, Y T Eunice Lo, Ulrika Maude, Nicholas J Timpson, Kate Northstone

**Affiliations:** 1 Cabot Institute for the Environment, University of Bristol, Bristol BS8 1UH, United Kingdom; 2 Elizabeth Blackwell Institute for Health Research, University of Bristol, Bristol BS8 1UH, United Kingdom; 3 Centre for Health, Humanities and Science, University of Bristol, Bristol BS8 1TB, United Kingdom; 4 MRC Integrative Epidemiology Unit at University of Bristol, Bristol BS8 2BN, United Kingdom; 5 Population Health Sciences, Bristol Medical School, University of Bristol, Bristol BS8 2BN, United Kingdom

**Keywords:** heat, heatwave, lived experience, climate change, ALSPAC

## Abstract

So far, there has been little evidence of the impact of extreme heat on behaviour and wellbeing in daily life, beyond gross health metrics like hospital admissions and mortality. Data are needed to better understand the relative impact of a changing climate across life course strata and ultimately inform effective adaptation strategies. Using the UK September 2023 heatwave as a case study, we surveyed 3242 participants from the Avon longitudinal study of parents and children about their lived experience of extreme heat. Responses evidenced perceived adverse impacts on sleep quality (67% of the participants), productivity at home (41%), appetite (29%), and productivity at work (25%). Beneficial impacts were perceived for mood (39% of the participants) and physical health (20%). Demographic (age and gender) and socio-economic variables (employment status and housing type) were associated with differences in the reported heat effects for lived experience of sleep quality, productivity at home and mood. Participants who were female, ⩽34 years of age, and unemployed/not working, perceived ‘a lot worse’ impacts on sleep quality in greater numbers than other sub-groups (age: 25% ⩽ 34 *versus* 10% ⩾ 65; gender: 21% female *versus* 10% male; employment status: 37% unemployed/not working *versus* 19% employed). These groups, as well as people living in flats, are also perceived ‘a lot and slightly worse’ impacts on productivity at home and mood, more often than other sub-groups. Despite the majority of the (82%) participants reporting awareness of the UK Health Security Agency and Met Office amber heat-health alerts, only 34% reported taking adaptive measures. Understanding the physiological and socio-economic drivers behind the experience of extreme heat is crucial for building resilience. Established cohort studies can be usefully employed to rapidly measure impact and variation in response.

## 1 Introduction

Extreme heat events are increasing in frequency and intensity as the consequences of anthropogenic climate change continue to unfold (Seneviratne *et al* 2021). Heatwaves present both direct and indirect risks to health and wellbeing (Watts *et al* 2015), as well as to healthcare delivery through service disruption and overheating (CCC 2021, Romanello *et al* 2023). Their increased likelihood is predicted to add to the global burden of heat-related disease (Cissé *et al* 2022, Romanello *et al* 2023). However, implementation of effective risk planning and adaptation strategies can minimise or even prevent adverse health outcomes (Ebi *et al* 2021).

In England, at present, heatwave mortality ranges between ~300 and 3000 deaths per year (2011–2022) (Lo *et al* 2022, UKHSA 2024a, 2024b). In the absence of adaptation, annual heat-related deaths could rise to ~21 000 in the 2070s under high emission RCP8.5 conditions (Murage *et al* 2024). Population ageing, however, is expected to have a greater impact on future mortality than temperature rises (Murage *et al* 2024). Increased mortality will be driven by a combination of climate change and demographic change, due to the continued ageing of the population and vulnerability of older adults to extreme temperatures. In the UK, hospital admissions for causes including respiratory and renal problems, as well as dementia, are known to increase at high temperatures (Arbuthnott and Hajat 2017, Gong *et al* 2022, Konstantinoudis *et al* 2022). Without adaptation of healthcare infrastructure, hospital bed occupancy is projected to increase significantly in the summer months under high greenhouse gas emission scenarios (Israelsson *et al* 2022). Socio-economic and health inequalities affect people’s exposure and resilience to extreme heat.

Mortality records and hospital admission data provide fundamental insights into heat-related disease burden, but little evidence exists on the impact of extreme heat on daily life and wellbeing. In the UK, the prevalence of air-conditioning in homes is reported to be less than 3% (UK Government 2023). A survey of public perceptions of heatwaves carried out by the British Red Cross reported that 60% of the participants (*n* = 2,000) had experienced adverse health impacts from hot weather, including headaches, dizziness and heat rash (British Red Cross 2021). Similarly, a survey of Nottingham residents (*n* = 500), following the summer 2022 heatwave, found that most participants had experienced adverse effects on physical (71%) and/or mental (55%) health (Ogunbode *et al* 2024). The study concluded that although the adverse effects were mostly sub-clinical and non-fatal, they had a notable negative impact on wellbeing and quality of life. Furthermore, many people in at-risk groupsfor example, outdoor workers and those aged over 75 do not consider themselves to be vulnerable to extreme heat (British Red Cross 2021) and may not, therefore, take action to protect themselves (British Red Cross 2023).

Reduced productivity due to high temperatures was identified as a risk to the UK (Climate Change Committee 2021), with heat stress estimated to decrease productivity across Europe by 1.6% by the 2080s (Szewczyk *et al* 2021). Overall, further adaptations to address ‘risks to human health, well-being and productivity from increased exposure to heat in homes and other buildings’ was one of the highest priorities in the most recent independent UK Climate Change Risk Assessment (Climate Change Committee 2021). Motivated by this priority need, more detailed evidence of the lived experience of heatwaves can offer insights into the development of public health advice and inform effective adaptation strategies.

The research reported here draws on a world-renowned birth cohort study, the ‘Avon Longitudinal Study of Parents and Children’ (ALSPAC), to understand the lived experience of heatwaves. This rich dataset has enabled ALSPAC to generate policy-relevant health research over the last three decades, including contributions to understanding COVID-19 infections (Northstone *et al* 2020, Smith *et al* 2021), booster vaccine effectiveness (Cheetham *et al* 2023) and long COVID (Thompson *et al* 2022).

Recognising the importance of viewing extreme weather as a health threat on a par with major crises like COVID-19 (Lo *et al* 2024), the work reported here constitutes a pilot study on a novel engagement with ALSPAC that gathers evidence on the lived experience of a recent heatwave. From 4 to 10 September 2023, the UK experienced seven consecutive days of temperatures that exceeded 30 °C in parts of the country, including maximum temperature anomalies of up to 14 °C above the average for the time of year (supplementary figure S1(a)) (Met Office 2023b). 10th September was the hottest day of 2023 (33.5 °C in Kent; figure S1(b)) and this was also the longest recorded heatwave in the UK for September. During this period, an amber heat-health alert (second highest warning level in England’s alerting system) was issued across eight of the nine regions of England (London, South East, South West, North West, East Midlands, West Midlands, East of England, Yorkshire and the Humber). North East England had a lower yellow alert. The heat-health alert system uses temperature thresholds which reflect the relationship between temperature and mortality, as well as considering impacts across the health and social care systems (UKHSA and Met Office 2023). An amber alert indicates the need for an ‘enhanced response’ due to the potential risk to the whole population in these regions, as well as impacts across the whole health service and other non-health sectors (UKHSA and Met Office 2023).

Here, we use this heatwave as a case study to understand the impact of extreme heat on aspects of daily life that are sub-clinical and challenging to unpack in mortality and hospital admission data, as well as the user perspective on healthcare system access and impact on productivity. Eight aspects of daily life were chosen to investigate participants’ physical health (physical health, appetite), mental health (sleep quality, mood), access to health services (access to general practitioner (GP) surgery, access to accident and emergency (A&E)) and productivity (at home and at work). We also investigate public awareness of the heat-health alert and the actions taken. Our results have implications for communicating heatwave risk and adaptation guidance to users, which constitute two key elements of the Adverse Weather and Health Plan for England (UKHSA 2023b).

## 2 Methods

### 2.1 ALSPAC

Pregnant women resident in Avon, UK, with expected delivery dates between 1 April 1991 and 31 December 1992 were invited to take part in the study (Boyd *et al* 2013, Fraser *et al* 2013). Of the 20 248 eligible, 14 541 pregnancies enrolled. Numbers were boosted from the age of 7 years (Northstone *et al* 2019, Major-Smith *et al* 2022) and 14 833 women, and their 3807 enrolled partners (Northstone *et al* 2023) and 14 901 children (alive at 1 year of age) have been followed ever since. Please note that the study website contains details of all available data through a fully searchable data dictionary and variable search tool (ALSPAC 2024).

### 2.2 Survey

A rapid digital survey was designed and received ethical approval from the ALSPAC Ethics and Law Committee and the Local Research Ethics Committee. To maintain confidentiality, to reduce costs and to provide a rapid response, data gathered during this pilot study were not linked to the cohort records of individuals. Informed consent for the use of data collected via questionnaires and clinics was obtained from participants following the recommendations of the ALSPAC Ethics and Law Committee at the time. Full text responses were scanned by the ALSPAC research team for personal identifiers, which were redacted to maintain confidentiality.

In early November 2023, this rapid survey was distributed to all participants in the ALSPAC cohort who had a valid email address (*n* = 13 871). Participants who were in England during the 4–10 September heatwave were invited to respond and given 14 d to do so. The first survey question focused on the impacts of the heatwave on eight aspects of daily life: physical health, sleep quality, appetite, mood, productivity at home, productivity at work and access to general practitioner (GP) and A&E services. Participants were asked to rate each aspect of daily life under heat-wave conditions as ‘a lot worse’, ‘slightly worse’, ‘no change’, ‘slightly better’, ‘a lot better’ or ‘not applicable (N/A)’. The following questions focused on participants’ awareness of the heat-health alert (yes/no), changes to daily activities made in response (yes/no and optional free-text), and any additional information participants felt to be relevant (optional free-text). Remaining questions gathered data on participant characteristics: age category (25–34, 35–44, 45–54, 55–64, 65–74, 75–84, 85+), gender (female, male, non-binary), employment status (employed, unemployed/not working, student, retired), housing type (detached/semi-detached, terrace, flat, bungalow) and residential area (urban, suburbs, rural). For each question, participants were also given the option of ‘prefer not to say’ and ‘other’ (free-text). A limited number of characteristics were focused on maintaining participant confidentiality and enabling a short, rapid survey methodology. The full survey questions are provided in supplementary material S2.

### 2.3 Analyses

**Figure 1:**
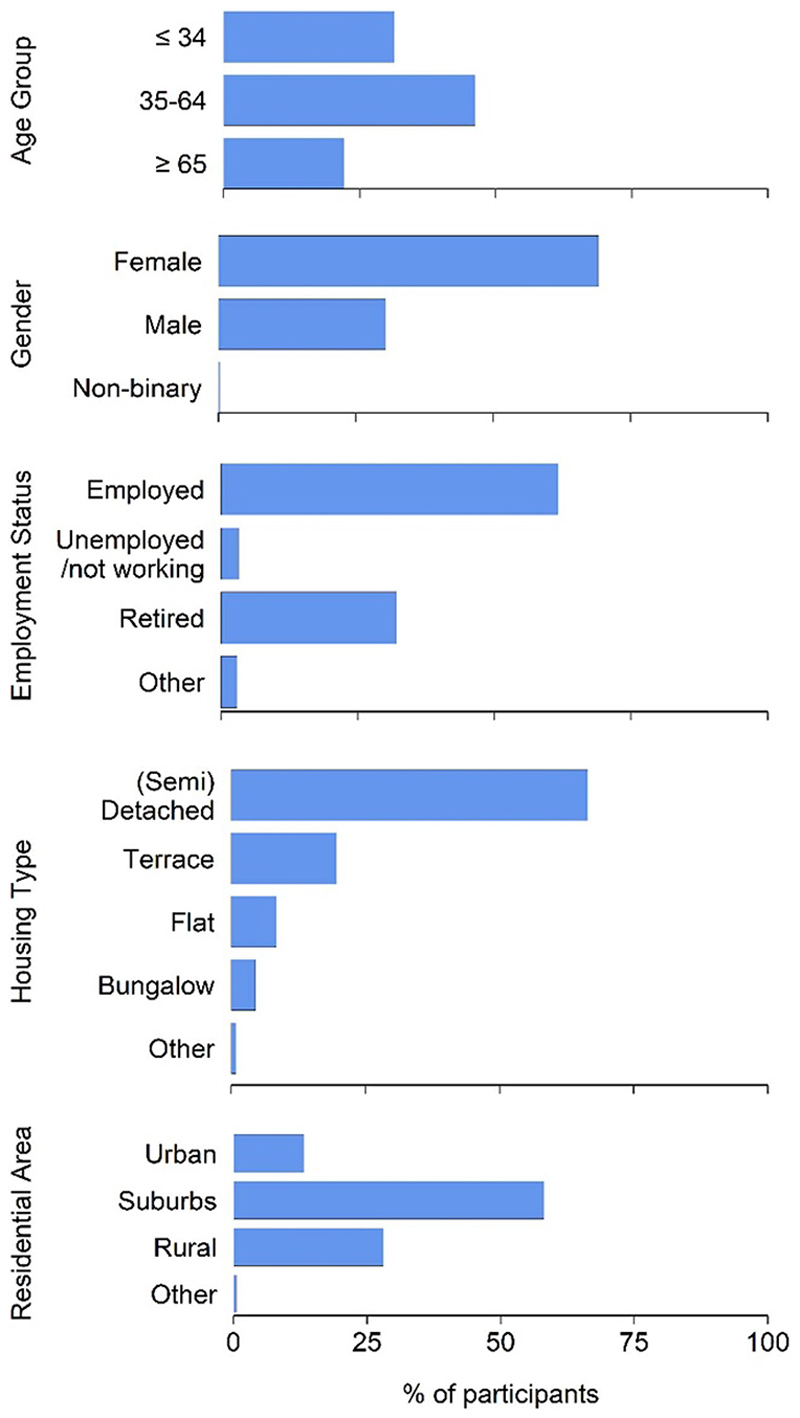
Summary of survey participant (*n* = 3,242) characteristics. Percentage (%) of total participants in age, gender, employment status, housing type and residential area, categories.

Statistical analyses and data visualisation were carried out in R 4.4.1 (R Core Team 2024). Gender groups are reported throughout as female, male or non-binary to maintain consistency with our survey question. We acknowledge this interchangeable use of sex (male/female) and gender (woman/man) terms as a limitation of this study, which is considered further in the discussion. Age categories were combined into three groups which better reflect the ALSPAC cohort composition and are also relevant for public health policy, ⩽34 years (children of the 90 s), 35–64 years (including parents of the children of the 90 s) and ⩾65 years (vulnerable age group in Adverse Weather and Health Plan (UKHSA 2023a)). For gender and socio-economic variables (employment status, housing type and residential area), categories with ⩽1% representation were presented in a summary of participants (figure 1) and then removed from subsequent analyses as the very small sample size prevented accurate comparison with other groups. Stacked bar charts and bivariate tables were used to explore and visually present perceived qualitative impacts of the heatwave (a lot worse, slightly better, no change, slightly better, a lot better, n/a), on the eight aspects of daily life investigated, as well as to examine trends across demographic and socio-economic sub-groups. For further visual assessment of age and gender groups, numerical values were assigned to qualitative descriptors (a lot worse = *−*2, slightly worse = *−*1, no change = 0, slightly better = 1, a lot better = 2) and mean values were calculated and plotted. Multinomial logistic regression was also used to assess whether demographic and socio-economic variables were more or less important in the lived experience of extreme heat. This approach aims to understand relationships in the data and should not be inferred as causality. This approach is presented to support analyses in supplementary materials S5 and S6.

Thematic analysis (Braun and Clarke 2006) was used to explore actions described by survey participants in free text responses, with qualitative coding carried out by the lead author based on discussions with the author team. Where multiple actions were taken by a single participant, each action was weighted (1/number of actions) to enable a quantitative approach to understanding changes to daily activities.

## 3 Results

A total of 3242 responses to the survey were analysed (response rate of 23%), with a summary of participant characteristics provided in figure 1 and a full breakdown in supplementary table S3. Due to the initial recruitment of pregnant women and their children into the ALSPAC study in the 1990 s, age and gender were not evenly distributed within the cohort (Northstone *et al* 2023) and this was reflected in the participants of this survey. Here, participants were 69% female, 30% male and 1% non-binary/preferred not to say. Across the age categories 32% were ⩽34 years, 46% were 35–64 years and 22% were ⩾65 years. Most participants were either employed (62%) or retired (32%), reported living in detached or semi-detached houses (67%) and either in suburban (58%) or rural (28%) areas.

### 3.1 Reported impacts on aspects of daily life

**Figure 2:**
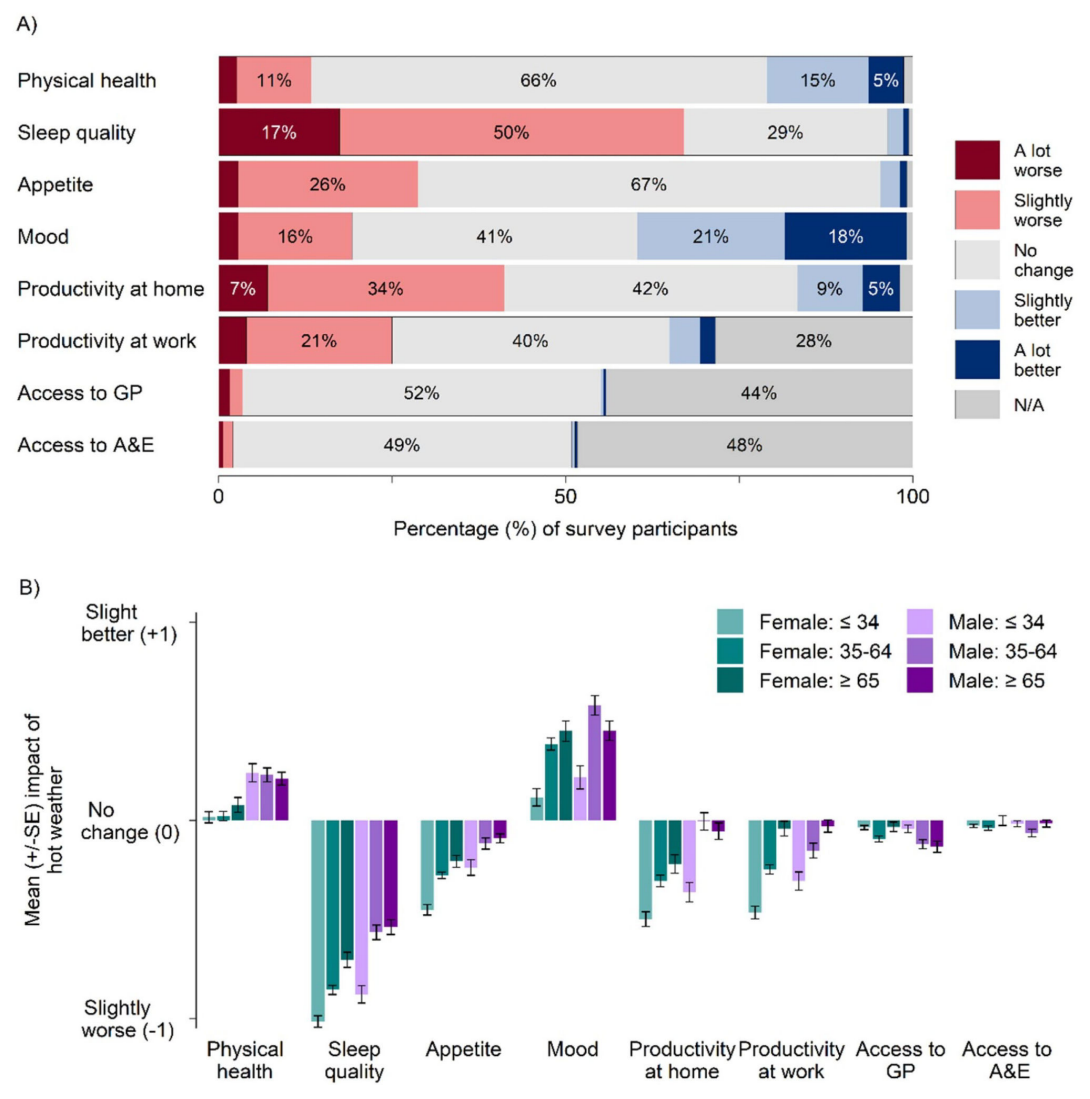
Impact of extreme hot weather on aspects of daily life of 3242 survey participants. A) Percentage of participants reporting adverse effects (a lot worse (dark red) and slightly worse (light red)), no change (pale grey), benefits (slightly better (light blue)) and a lot better (dark blue)), or N/A (dark grey). B) Mean (±SE) impact of hot weather on aspects of daily life, split by age (⩽34, 35–64, ⩾65) and gender (female, male) categories.

Sleep quality was the aspect of daily life perceived to be adversely impacted by the greatest number of participants, with 67% reporting it to be either ‘slightly’ or ‘a lot’ worse. Productivity at home (41%), productivity at work (25%) and appetite (29%) were also perceived as being adversely impacted (‘slightly’ or ‘a lot’ worse), although most participants felt that there was no change to these aspects of daily life. Mood and physical health, on the other hand, were perceived by 39% and 20% respectively, to be positively impacted (‘slightly’ or ‘a lot’ better), although the majority reported no change in mood or physical health (figure 2(a)).

**Figure 3:**
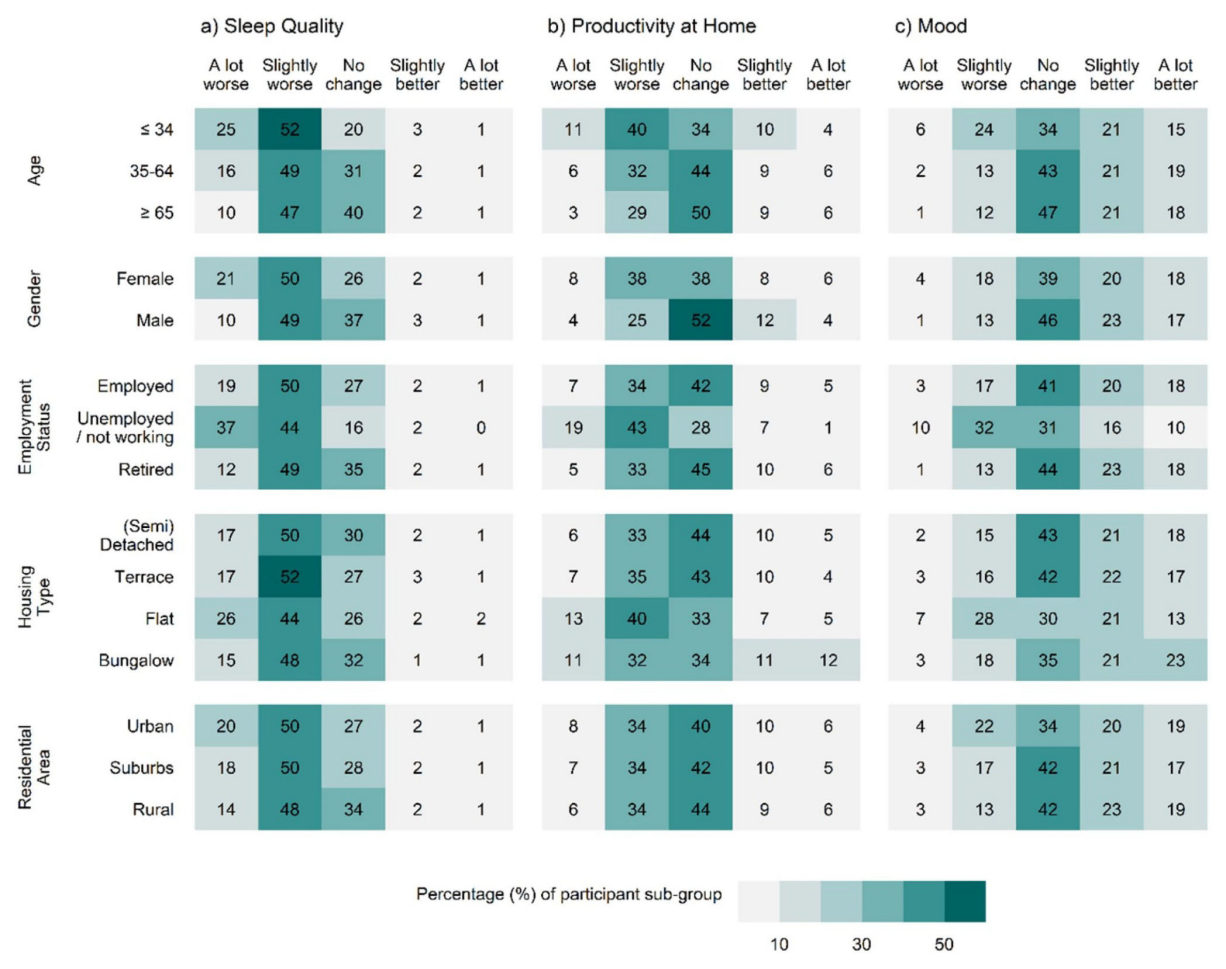
Perceived outcome of extreme hot weather on three aspects of daily life with the largest reported impacts (a) sleep quality, (b) productivity at home and (c) mood, by demographic (age and gender) and socio-economic (employment status, housing type and residential area) sub-groups. Percentage of participants within each sub-group perceiving the impact of extreme heat (a lot worse, slightly worse, no change, slightly better, a lot better).

Figure 2(b) suggests female participants and the youngest age group reported adverse outcomes more often than other groups, across multiple aspects of daily life: sleep, appetite, productivity at home and productivity at work. Further investigation of differences across all demographic (gender and age), and socio-economic (employment status, housing type and residential area) sub-groups focused on sleep quality, productivity at home and mood as aspects of daily life perceived to be impacted by the largest number of participants (figure 3).

Participants who were female, ⩽34 years of age, unemployed/not working, and living in flats perceived ‘a lot worse’ impacts on sleep quality in greater numbers than other sub-groups (age: 25%⩽ 34 *versus* 10% ⩾ 65; gender: 21% female *versus* 10% male; employment status: 37% unemployed/not working *versus* 19% employed; housing type: 26% flat *versus* 17% (semi)detached). Productivity at home was also reported to be ‘worse’ (a lot and slightly worse combined) more often by the same sub-groups (age: 51% ⩽ 34 *versus* 32% ⩾ 65; gender: 46% female *versus* 29% male; employment status: 62% unemployed/not working *versus* 41% employed; housing type: 53% flats *versus* 39% (semi)detached). While mood was perceived by many participants (39%) to be ‘better’, there were no clear differences between the sub-groups. However, participants ⩽34 years of age, unemployed/not working and those living in flats, appear to perceive ‘worse’ impacts on mood more frequently than other sub-groups (age: 30% ⩽ 34 *versus* 13% ⩾ 65; employment status: 42% unemployed/not working *versus* % 20 employed; housing type: 35% flats *versus* 17% (semi)detached). No clear differences were found between residential area subgroups. A full breakdown of the percentage of participants reporting perceived impacts, across all eight aspects of daily life, is provided in supplementary figure S4.

A multinomial logistic modelling approach to investigating differences between sub-groups, presented in supplementary analysis S5, adds to the evidence that females, the youngest age group, people who are unemployed or not working and those living in flats, are more likely to report experiencing adverse outcomes across sleep, productivity at home and mood than other groups (supplementary table S6).

### 3.2 Physical and mental health symptoms

A number of survey participants (*n* = 496) also provided information on the physical and mental health impacts of the free text responses. The most frequently reported impacts (see table 1), i.e. improved mood/enjoyed the weather; lethargy/-fatigue; and poor sleep quality corroborated the findings of earlier survey questions (see figure 2). However, frequent mentions of anxiety, such as ‘our homes are not built for this weather and it is worrying’; poor mood, for instance, ‘Fed up of being in a hot flat in extreme weather’; and irritability suggest significant adverse effects of extreme heat on mental health and wellbeing not captured by the main survey questions. Anxiety was regularly mentioned alongside concern for young children: ‘Worrying to keep a young baby cool’. Another source of anxiety was the wellbeing ofrelatives: ‘Concerned for elderly parent and baby granddaughter to ensure they were ok’. Yet another notable concern was triggered by climate anxiety: ‘It was worrying. Climate change is real. Nothing seems to be changing’. The heatwave also had an adverse impact on pregnancy and the menopause. One participant reported that ‘pregnancy related water retention increased’, while another wrote that ‘in the grip of the menopause many of those symptoms were increased … making things even more uncomfortable’ (table 1). This also applied to exacerbated symptoms of existing or chronic health conditions, including respiratory conditions: ‘Made my asthma worse’; metabolic conditions: ‘Diabetes felt harder to control’; and autoimmune conditions: . . with Multiple Sclerosis, it is always a bit more stressful. More fatigue and more flare-ups in the heat’. Some participants, however, experienced beneficial effects from the heatwave, including one respondent suffering from arthritis, whose ‘joints did not ache as much in the warmer weather’.

### 3.3 Awareness of heat-health alerts and adaptation

**Figure 4:**
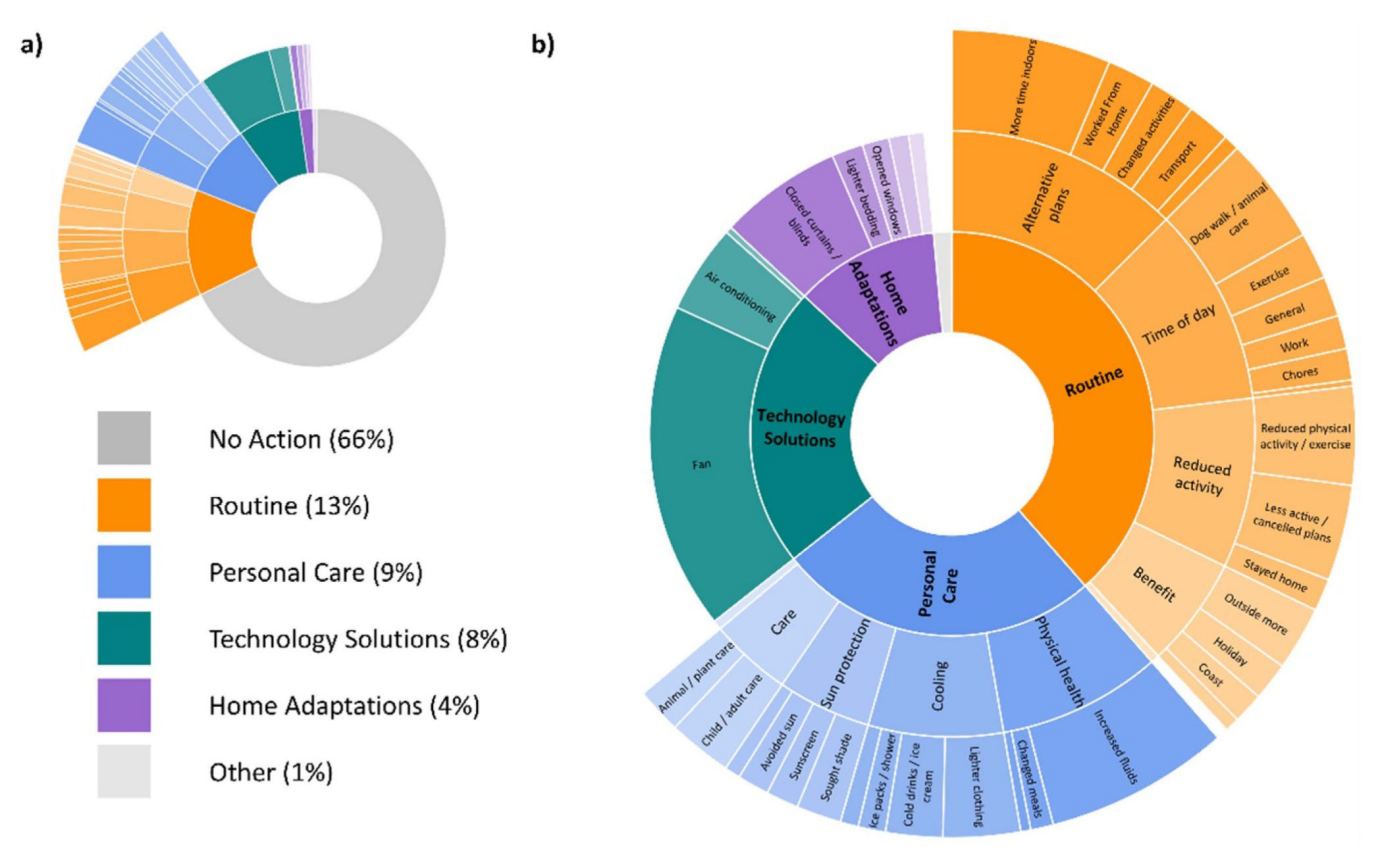
Changes to daily activities in response to heat-health warnings and extreme hot weather, (a) adaptive behaviours as a percentage of the total, grouped by major themes and (b) adaptive behaviours as a percentage of the total, excluding no action.

82% of survey participants were aware of the heat-health alert issued ahead of the hot weather. Changes to daily activities were described in free text responses by 34% of the survey participants and these were categorised into four main areas of action: changes in routine (13%), personal care (9%), technology solutions (8%) and home adaptations (4%). A visual summary of the changes made under each of these categories is shown in figure 4(b). Changes in routine included making alternative plans, changing the time of day for activities such as dog walking, reducing certain activities and taking advantage of the hot weather. Personal care actions included taking care of one’s physical health, cooling the body, using sun protection, and caring for others, such as elderly relatives. Technology solutions involved turning on fans and air conditioners, whereas home adaptations involved drawing curtains, using lighter bedding and opening windows. A full breakdown of all adaptive behaviours in each category as well as the number of participants reporting them is provided in supplementary table S7.

**Figure 5:**
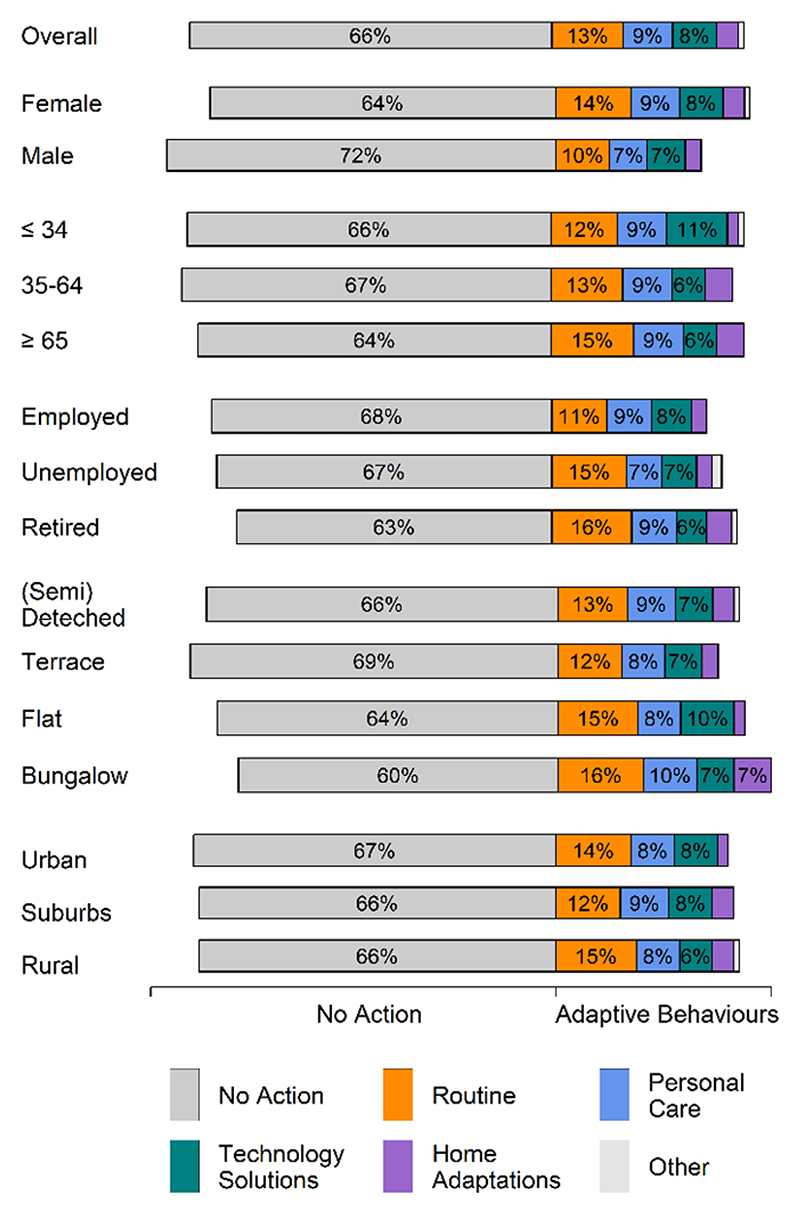
Breakdown of actions taken in response to heat-health warnings and extreme hot weather by participant groups, compared to overall.

Breaking down these behaviours by demographic and socio-economic groups (figure 5) suggests that females were more likely to take action or report taking action, than males. Females also appeared to be more likely to make changes to ‘routine’ than males. In addition, participants ⩽34 years of age and those living in flats were more likely to make use of ‘technology solutions’, and less likely to make ‘home adaptations’ compared to the older age groups and those living in bungalows (see supplementary table S7).

## 4 Discussion

Capturing evidence of the lived experience of extreme heat has provided insights beyond established heat-health burden metrics and uncovered trends across major demographic and socio-economic groups. Despite the perceived benefits to mood and physical health reported by participants in this study (figure 2(a)), overall, the impacts of extreme heat on the aspects of daily life investigated were adverse and included direct, albeit sub-clinical, physical and mental health impacts. Sleep quality, productivity at home and appetite were perceived to be most adversely impacted and our study revealed strong evidence to suggest that age, gender, employment status and housing type are all associated with adverse impacts on these key aspects of daily life. To our knowledge, this is the largest survey of England-based residents which has sought to understand the lived experience of extreme heat, and the first to consider variation across demographic and socio-economic groups. Understanding the social determinants of vulnerability to extreme heat in the public will be vital in minimising health risks (UKHSA 2023a) in England.

Our findings confirm the results of a smaller survey of lived experience in Nottingham, where residents reported, ‘difficulty sleeping’ as the most frequent (71%) health impact of extreme heat (Ogunbode *et al* 2024). Data gathered via sleep-tracking wristbands worn by participants across 68 countries and linked to local meteorological data, also suggests that warmer temperatures are shortening sleep duration globally (Minor *et al* 2022). Sleep is a vital component of good health and both short-term and chronic sleep disruptions are associated with negative impacts on physical health, mental health, productivity and safety (Foster 2020, Ramar *et al* 2021). Crucially, sleep disruption has been suggested as a possible pathway for exacerbation of poor mental health during extreme heat (Lõhmus 2018). There is growing evidence of an association between heatwaves and poor mental health (Rony and Alamgir 2023, Thompson *et al* 2023), and mitigating sleep disruption may be a useful tool in addressing this issue.

Despite the increased vulnerability of older age groups to extreme temperatures in mortality data (Murage *et al* 2024), our survey data suggest ⩾65 s are reporting fewer perceived adverse impacts on daily life compared to younger age groups. Explanations for this could include lower health risk perception and socio-economic factors. There is evidence that half of older adults (⩾65 s) in England do not consider their health to be at risk in hot weather, due to not self-identifying as ‘vulnerable’, past experiences, and stoicism (Turner *et al* 2024). A serious concern for public health is that those at risk may also be the ones who do not recognise the risk and therefore are not making behavioural changes to support thermoregulation (Millyard *et al* 2020). However, over 65 s are also less likely to be in full-time employment or to have childcare responsibilities, which may enable them to be more flexible and adaptive in their behaviours. Data presented here on actions taken in response to the heatwave, do not suggest that ⩾65 s are more (or less) likely to make use of adaptive behaviours than younger age groups (figure 5).

The data presented here suggests gender is also an important variable in the lived experience of extreme heat, with female participants reporting adverse impacts on sleep quality, productivity at home and mood more often than males. In the UK, domestic work and childcare are still predominantly carried out by women (National Centre for Social Research 2023) and are likely to be an important driver of gender differences in perceived impacts on aspects of daily life. As already acknowledged, the interchangeable use of sex and gender terms is a limitation of this study. Further steps during data collection are required to determine whether differences exist across both sex and gender groups (Kaufman *et al* 2023).

A benefit to mood was reported in direct survey questions (see figures 2 and 3) and reflected in free text responses (see table 1); however, poor mood, irritability and anxiety, were also mentioned by a number of individuals. Among these, the most frequent reasons provided, were concerns for young children or elderly relatives, followed by ‘climate anxiety’. These findings provide further evidence that the adverse impacts of extreme heat on mental health are both direct and indirect. Those who are not directly physiologically vulnerable may often be caring for people who are, therefore, greater awareness of adaptive behaviours that support thermoregulation can support good mental health across demographic groups.

A crucial finding of this study is that currently, adaptive behaviours are only carried out by a minority (34%) of the surveyed UK public. Whilst this is information based on a sub-set of contributing responders, a priority for public health advice will be to raise awareness of the options available. Data presented here do suggest that both older and younger members of the population, females, people who are unemployed/not working, and those living in flats could benefit from the guidance.

Working with an established cohort for this pilot study offered the ability to rapidly measure perceived impact, and variation in response, to extreme hot weather. Future studies with this cohort offer the opportunity to address some limitations in the data gathered, by connecting through to the full ALSPAC dataset, which includes participants’ residential address, NHS record, clinical measurements and biological samples, and characterise a broader group. However, it is important to note the likely bias in survey participants when using an existing cohort. The age and gender breakdown of participants in this survey reflects the overall make-up of the ALSPAC study (Northstone *et al* 2023) but not of the wider UK population. Previous studies have shown that ALSPAC mothers are more likely to live in owner-occupied accommodation, to have a car, and to be white, compared to the Avon or UK average (Fraser *et al* 2013). Their children were less likely to be eligible for free school meals and have higher educational attainment than the national average (Boyd *et al* 2013). Other health cohorts have similar selection biases (Fry *et al* 2017). Most recent data held show 70% of the cohort to have a postcode in the South West region of England (personal correspondence with the ALSPAC cohort team); future surveys should address this limitation in the geographic location of participants to fully understand the impact of extreme weather events. We acknowledge that our sample is not fully representative of the UK population, this means that our results may be underestimates of the wider UK public lived experience. Expanding this research to a more diverse population across the UK and beyond will be an important next step for this type of research.

A further limitation in our rapid survey approach was the use of a qualitative scale which will inevitably result in variation in individuals’ interpretation of ‘slightly’ or ‘a lot’ better/worse. We did not ask our participants to define what they meant in order to calibrate their responses, and this will need to be considered for future surveys. In addition, our survey was carried out two months after the heatwave period which may lead to recall bias. The UK’s September 2023 heatwave occurred at the end of the warm season, following a July that was cooler than the 1991–2020 average and the sixth wettest on record (Met Office 2023a), and an August involving two named storms. The September hot weather, therefore, could have been welcome news for some, affecting the mood of participants more positively than an equivalent heatwave in a different context. Future work deploying similar surveys about heatwaves in other months or of different intensities is recommended to gain a full picture of the lived experience of extreme heat.

## 5 Conclusion

The data presented here provide important insights into perceived daily life impacts of extreme heat and point to the pressing need for continued awareness-raising of risks and adaptive measures to minimise reported adverse impacts during heatwaves. Younger adults; females; people who are unemployed/not working; and those living in flats; are experiencing adverse impacts on key aspects of daily life which could be alleviated by improved knowledge of, and support for, effective adaptive measures. These groups need to be made aware of risks as well as adaptive behaviours that can support thermoregulation and prevent heat-related adverse impacts on health and wellbeing. On the other hand, over 65 year-olds, who have previously been shown to be more vulnerable to extreme heat, appear less likely to perceive ‘worse’ impacts and may benefit from awareness raising of risks.

## Supplementary Material

Supplementary material for this article is available online

Supplementary Materials

## Figures and Tables

**Table 1:** Most frequently reported physical and mental health symptoms mentioned in free text responses.

	Physical or mental health symptom	Count
1	Mood improved/Enjoyed the weather	196
2	Lethargy/Fatigue	56
3	Deterioration in sleep quality	35
4	Anxiety	34
5	Poor mood/Irritability	30
6	Discomfort	20
7	Breathing difficulties + Asthma worse	19
8	Menopause symptoms worse	11
9	Swollen feet/ankles	7
10	Pregnancy more difficult	6

## Data Availability

The data that support the findings of this study will be available by request to the authors.
