# Supplementary material for: Extreme heat impacts on daily life and adaptive behaviours captured through lived experience
Source: Environ Res Lett. Author manuscript; Available in PMC 2026 Apr 22. (PMC7619029; doi:10.1088/1748-9326/adcbc5)
Supplement: Supplementary Materials [file EMS213298-supplement-Supplementary_Materials.pdf]

Extreme heat impacts on daily life and adaptive behaviours captured through lived experience

Joanne L. Godwin[1], Y.T. Eunice Lo[1,2], Ulrika Maude[3], Nicholas J. Timpson[4, 5] and Kate Northstone[5]

[1] Cabot Institute for the Environment, University of Bristol, Bristol, BS8 1UH, UK
[2] Elizabeth Blackwell Institute for Health Research, University of Bristol, BS8 1UH, UK
[3] Centre for Health, Humanities and Science, University of Bristol, Bristol, BS8 1TB, UK
[4] MRC Integrative Epidemiology Unit at University of Bristol, Bristol BS8 2BN, UK
[5] Population Health Sciences, Bristol Medical School, University of Bristol, Bristol BS8 2BN, UK

Supplementary Materials

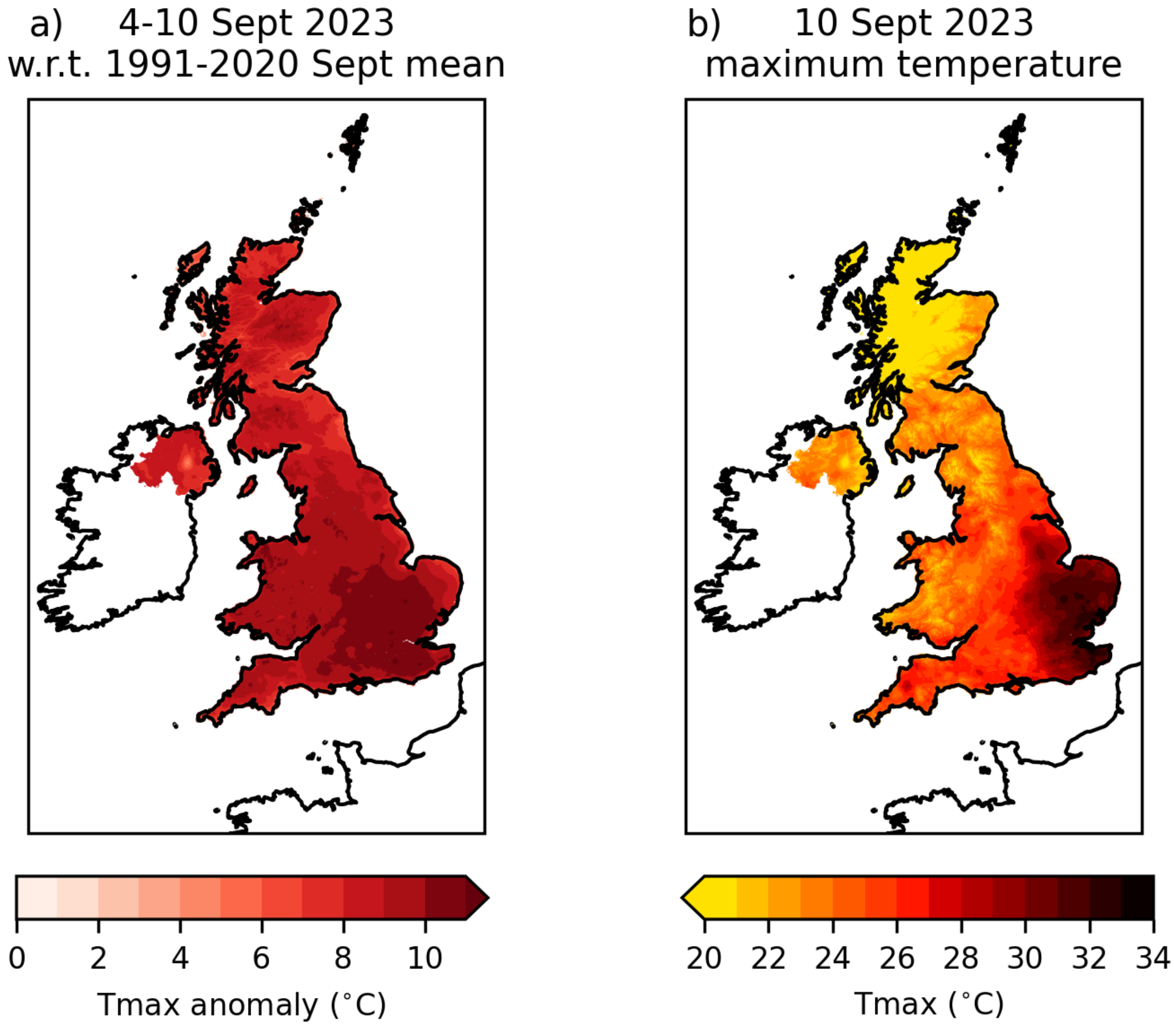

Supplementary Figure S1 | Daily maximum temperature (Tmax) across the UK for a) the duration of the heatwave (4th-10th September 2023), plotted here as temperature anomalies with respect to the average 1991-2020 September daily maximum temperature; and b) the hottest day (10th September 2023). All data here are from the UK Met Office HadUK-Grid dataset (Hollis *et al.*, 2019).

Hollis, D., McCarthy, M., Kendon, M., Legg, T. and Simpson, I., 2019. HadUK-Grid—A new UK dataset of gridded climate observations. *Geoscience Data Journal*, 6(2), pp.151–159. https://doi.org/10.1002/gdj3.78

Supplementary Material S2 | Survey Questions

The following text and questions were presented in a Microsoft Form. A link was sent to participants via email.

Lived experience of recent hot weather survey

Introduction

We are interested in understanding how you might be affected by extreme weather events. For some people, a particularly wet day might bring a minor inconvenience; for others, it can be hugely disruptive. In September, we experienced a period of particularly warm weather for the time of year and would like to ask you some questions about how you coped during this period if you can recall.

Please remember that this survey is completely anonymous; we will not be able to link your responses to any other data you have given to us as part of Children of the 90s. If you provide any personal information in any of the free text responses, this will be anonymised before the survey results are shared with any researchers.

About the hot week in England in September 2023

We would like you to think back to the days between 4th September and 10th September 2023, during which the UK experienced 30 degree Celsius heat for seven consecutive days. If you were not in England during this period the survey will not apply to you. We understand this may be difficult to remember, but all your response are valuable.

1. How were different aspects of your life affected by this period of hot weather, compared to normal?

	A lot worse	Slightly worse	No change	Slightly better	A lot better	N/A
Mood						
Productivity at home						
Productivity at work						
Sleep quality						
Appetite						
Physical health						
Access to GP						
Access to hospital or emergency care						

2. Were you aware of the amber Heat-Health Alert during the hot week? Yes | No

3. Did you make any changes to your usual daily activities in order to deal with the heat? For example, work from home, change your usual mode of transport, cancel personal or professional appointments, buy a fan.

   a. I made changes in response to the heat warning (i.e. in advance of heat wave) Yes | No
   If yes, please tell us, what changes you made? Free text

   b. I made changes in response to the weather itself Yes | No
   If yes, please tell us, what changes you made? Free text

   c. I made changes for some other reasons Yes | No
   If yes, please tell us, what changes you made Free text

4. Is there anything else you would like to tell us about how you were affected by this period of particularly warm weather? Free Text

About you

5. What is your gender?
   Male | Female | Non-binary | Prefer not to say | Other (text box)

6. What is your age?
   25-34 | 35-44 | 45-54 | 55-64 | 65-74 | 75-84 | 85+ | Prefer not to say

7. What type of housing do you live in?
   Flat | Detached or semi-detached house | Terraced house | Bungalow | Prefer not to say | Other (text box)

8. How would you describe the area where you live?
   City or town centre | City or town suburbs| Rural/Countryside | Prefer not to say | Other (text box)

9. What is your job status?
   Employed | Unemployed or not working | Student | Retired | Prefer not to say | Other (text box)

10. I understand that by submitting the information I have provided above, my data cannot be identified and attributed to me and therefore cannot be deleted. It will not be made available with the rest of my Children of the 90s data as it cannot be linked. Tick box to indicate

Supplementary Table S3 | Survey participant characteristics (total = 3,242).

Characteristic	Survey categories		n	%	Model levels	n	%
**Gender**	Female		2241	69	Female	2241	69
	Male		987	30	Male	987	31
	Non-binary		8	0.5	Other	removed	
	Prefer not to say		6	0.5			
**Age**	≤ 34		1021	31	≤ 34	1021	32
	35 – 44		2	0	35 – 64	1502	46
	45 – 54		87	3			
	55 – 64		1413	44			
	65 – 74		684	21	≥ 65	717	22
	75 - 84		33	1			
	Prefer not to say		2	0	Other	removed	
**Employment**	Employed		1869	58	Employed	2003	64
	Self-employed		134	4			
	Unemployed / not working		104	3	Unemployed or not working	104	3
	Retired		1038	32	Retired	1038	33
	Other	Maternity leave	20	1	Other	removed	
		Carer	16	0.5			
		Ill health / disability	15	0.5			
		Student	14	0			
		Volunteer	7	0			
	Prefer not to say		25	1			
**Housing Type**	(Semi) Detached		2158	67	(Semi) Detached	2158	67
	Terrace		634	20	Terrace	634	20
	Flat		276	9	Flat	276	9
	Bungalow		145	4	Bungalow	145	5
	Other	Caravan / Motorhome	7	0	Other	removed	
		Park home	5	0			
		Homeless	3	0			
		Other	7	0			
	Prefer not to say		7	0			
**Residential Area**	Urban		429	13	Urban	429	13
	Suburbs		1886	58	Suburbs	1886	59
	Rural		888	27	Rural	909	28
	Coast		21	1			
	Other		18	1	Other	removed	

Supplementary Table S4 | Bivariate tables for eight aspects of daily life investigated. Percentage (%) of each sub-group reporting perceived impacts across five qualitative descriptors (a lot worse, slightly worse, no change, slightly better, a lot better) and collapased levels to compare perceived 'worse' (a lot and slightly worse) and 'better' (a lot and slightly better) impacts against no change.

Aspect of daily life	Variable	Category	Percentage (%) of participants in sub-groups							
			Perceived impact					Collapsed levels		
			A lot worse	Slight worse	No change	Slightly better	A lot better	Worse	No change	Better
**Physical health**	Age	< 34	3	11	64	16	5	14	64	21
		35-64	3	12	65	13	6	14	65	19
		65+	2	8	69	15	5	9	69	20
	Gender	Female	3	13	65	13	5	16	65	18
		Male	1	5	68	19	5	6	68	24
	Employment status	Employed	2	10	66	15	5	12	66	20
		Unemployed	13	22	51	10	2	35	51	12
		Retired	2	10	67	15	5	12	67	20
	Housing type	(Semi) Detached	2	10	67	15	5	12	67	20
		Terrace	3	10	66	14	6	13	66	20
		Flat	5	13	63	13	5	18	63	19
		Bungalow	6	15	54	15	7	21	54	22
	Residential area	Urban	3	11	63	15	6	14	63	21
		Suburbs	3	11	66	15	5	13	66	19
		Rural	2	10	66	15	6	13	66	20
**Sleep quality**	Age	< 34	25	52	20	3	1	77	20	3
		35-64	16	49	31	2	1	65	31	3
		65+	10	47	40	2	1	57	40	3
	Gender	Female	21	50	26	2	1	70	26	3
		Male	10	49	37	3	1	59	37	3
	Employment status	Employed	19	50	27	2	1	69	27	3
		Unemployed	37	44	16	2	0	81	16	2
		Retired	12	49	35	2	1	61	35	3
	Housing type	(Semi) Detached	17	50	30	2	1	66	30	3
		Terrace	17	52	27	3	1	69	27	3
		Flat	26	44	26	2	2	70	26	4
		Bungalow	15	48	32	1	1	63	32	3
	Residential area	Urban	20	50	27	2	1	70	27	3
		Suburbs	18	50	28	2	1	69	28	3
		Rural	14	48	34	2	1	62	34	3

Supplementary Table S4 continued

Aspect of daily life	Variable	Category	Percentage (%) of participants in sub-groups							
			Perceived impact					Collapsed levels		
			**A lot worse**	**Slight worse**	**No change**	**Slightly better**	**A lot better**	**Worse**	**No change**	**Better**
**Appetite**	Age	< 34	6	34	54	4	1	40	54	5
		35-64	2	23	71	2	1	25	71	3
		65+	1	19	75	3	1	20	75	4
	Gender	Female	3	29	63	2	1	33	63	3
		Male	1	18	75	4	1	20	75	5
	Employment status	Employed	3	27	65	3	1	30	65	4
		Unemployed	12	39	44	2	2	51	44	4
		Retired	1	22	74	2	1	23	74	3
	Housing type	(Semi) Detached	2	25	69	2	1	27	69	3
		Terrace	3	28	63	4	1	30	63	6
		Flat	8	32	53	5	1	40	53	6
		Bungalow	3	18	72	3	2	21	72	6
	Residential area	Urban	3	29	63	3	1	33	63	3
		Suburbs	3	26	66	3	1	29	66	4
		Rural	2	24	69	3	1	26	69	4
**Mood**	Age	< 34	6	24	34	21	15	30	34	36
		35-64	2	13	43	21	19	15	43	40
		65+	1	12	47	21	18	13	47	40
	Gender	Female	4	18	39	20	18	22	39	38
		Male	1	13	46	23	17	13	46	40
	Employment status	Employed	3	17	41	20	18	20	41	38
		Unemployed	10	32	31	16	10	41	31	26
		Retired	1	13	44	23	18	14	44	41
	Housing type	(Semi) Detached	2	15	43	21	18	17	43	39
		Terrace	3	16	42	22	17	19	42	39
		Flat	7	28	30	21	13	35	30	34
		Bungalow	3	18	35	21	23	21	35	43
	Residential area	Urban	4	22	34	20	19	26	34	39
		Suburbs	3	17	42	21	17	19	42	38
		Rural	3	13	42	23	19	15	42	41

Supplementary Table S4 continued

Aspect of daily life	Variable	Category	Percentage (%) of participants in sub-groups							
			Perceived impact					Collapsed levels		
			A lot worse	Slight worse	No change	Slightly better	A lot better	Worse	No change	Better
**Productivity at home**	Age	< 34	11	40	34	10	4	52	34	14
		35-64	6	32	44	9	6	38	44	15
		65+	3	29	50	9	6	32	50	15
	Gender	Female	8	38	38	8	6	46	38	14
		Male	4	25	52	12	4	29	52	16
	Employment status	Employed	7	34	42	9	5	42	42	15
		Unemployed	19	43	28	7	1	63	28	8
		Retired	5	33	45	10	6	37	45	15
	Housing type	(Semi) Detached	6	33	44	10	5	39	44	15
		Terrace	7	35	43	10	4	42	43	14
		Flat	13	40	33	7	5	54	33	12
		Bungalow	11	32	34	11	12	43	34	23
	Residential area	Urban	8	34	40	10	6	42	40	16
		Suburbs	7	34	42	10	5	42	42	15
		Rural	6	34	44	9	6	40	44	15
**Productivity at work**	Age	< 34	8	34	42	7	2	42	42	9
		35-64	3	20	43	4	3	23	43	6
		65+	1	5	30	2	2	6	30	4
	Gender	Female	5	22	39	4	2	27	39	6
		Male	2	18	43	6	2	21	43	8
	Employment status	Employed	6	31	50	6	3	36	50	9
		Unemployed	4	20	27	1	0	24	27	1
		Retired	0	4	21	1	1	4	21	2
	Housing type	(Semi) Detached	3	19	39	4	2	22	39	6
		Terrace	5	24	43	5	3	29	43	8
		Flat	8	33	40	7	3	41	40	10
		Bungalow	5	20	34	6	1	25	34	7
	Residential area	Urban	7	27	42	7	3	34	42	10
		Suburbs	4	21	40	5	2	25	40	7
		Rural	3	19	40	3	2	22	40	5

Supplementary Table S4 continued

Aspect of daily life	Variable	Category	Percentage (%) of participants in sub-groups							
			Perceived impact					Collapsed levels		
			**A lot worse**	**Slight worse**	**No change**	**Slightly better**	**A lot better**	**Worse**	**No change**	**Better**
**Access to GP**	Age	< 34	1	2	67	0	0	3	67	1
		35-64	2	1	47	1	0	4	47	1
		65+	1	2	38	0	1	3	38	1
	Gender	Female	2	2	53	0	0	3	53	1
		Male	2	2	50	0	0	4	50	0
	Employment status	Employed	2	2	59	0	0	3	59	0
		Unemployed	4	7	56	1	2	11	56	3
		Retired	1	2	35	0	1	3	35	1
	Housing type	(Semi) Detached	1	2	49	0	0	3	49	1
		Terrace	2	2	55	0	0	4	55	0
		Flat	2	3	63	1	1	5	63	1
		Bungalow	6	2	52	1	1	8	52	1
	Residential area	Urban	1	3	56	0	1	4	56	1
		Suburbs	2	2	51	1	0	4	51	1
		Rural	1	2	50	0	0	3	50	1
**Access to A&E**	Age	< 34	0	1	66	0	0	2	66	0
		35-64	1	2	44	1	0	2	44	1
		65+	1	1	34	0	1	2	34	1
	Gender	Female	1	1	50	1	0	2	50	1
		Male	1	2	47	0	0	2	47	1
	Employment status	Employed	1	1	57	1	0	2	57	1
		Unemployed	1	4	55	1	1	5	55	2
		Retired	1	1	30	0	1	2	30	1
	Housing type	(Semi) Detached	0	1	46	0	0	2	46	1
		Terrace	1	2	53	1	0	3	53	1
		Flat	1	2	61	1	1	3	61	2
		Bungalow	3	2	50	1	1	5	50	1
	Residential area	Urban	0	2	55	1	1	2	55	1
		Suburbs	1	1	49	1	0	2	49	1
		Rural	1	1	46	0	0	2	46	0

Supplementary Analysis S5

S5.1 Methods

Multinomial logistic regression models, constructed using the 'nnet' package (Venables & Ripley, 2002), were used to assess the influence of demographic (gender and age), and socio-economic (employment status, housing type and residential area) variables, on aspects of daily life impacted by the heatwave. Multinomial logistic regression breaks the outcome variable down into a series of comparisons between two categories, one of which is a reference level (Field *et al.*, 2012). To explore overarching trends in the data, qualitative descriptions of impacts on aspects of daily life were categorised to compare 'worse' impacts, ('slightly' and 'a lot' worse combined) and 'better' impacts, ('slightly' and 'a lot' better combined), to 'no change', which was set as the baseline level. Models were fitted using maximum likelihood, and stepwise deletions were made to reach a minimum adequate model. During deletions, likelihood ratio tests and AIC values were used to compare models with and without demographic and socio-economic covariables (Crawley, 2013). The effect of groups within gender (female (reference level) *versus* male), age (≤34 (reference level), *versus* 35-64 and ≥65), employment status (employed (reference level), *versus* unemployed or not working, and retired), housing type (detatched or semi-detached (reference level), *versus* terrace, flat and bungalow), and residential area (urban (reference level), *versus* suburbs, and rural)), were assessed using z-statistics and odds ratios (OR) (Field *et al.*, 2012).

S5.2 Results

Statistical analysis to further investigate the influence of gender and age, as well as socio-economic factors (employment status, housing type and residential area) focused on sleep quality, productivity at home and mood as the aspects of daily life with the largest reported impacts. The full outputs of best fit multinomial logistic regression models are provided in Table 1.

Adverse impacts ('worse' *vs.* no change), on sleep quality were associated with the gender, age and employment status of participants (Table 1). Being female was associated with an increased odds (OR (95% CI)) of reporting 'worse' sleep quality (OR = 1.62 (1.37, 1.92), $p < 0.001$), compared to males. Being either ≤34 or 35-64 years in age, compared to ≥65, was also associated with increased odds of reporting 'worse' sleep quality (≤34: OR = 2.78 (2.12, 3.66), $p < 0.001$; 35-64: OR = 1.39 (1.12, 1.73), $p < 0.001$). Compared to participants who were employed, those who were unemployed / not working, also had an increased odds of reporting 'worse' sleep (OR = 2.03 (1.15, 3.58), $p = 0.01$).

Impacts on productivity at home were also associated with gender, age and employment status, as well as housing type (Table 1). Females were more likely to report productivity at home to be 'worse' compared to males (OR = 2.12 (1.77, 2.53), $p < 0.001$). Being in either of the younger age groups, compared to ≥65 years of age, was associated with an increased odds of reporting 'worse' productivity at home (≤34: OR = 1.38 (1.09, 1.73), $p = 0.01$ and 35-64: OR = 2.84 (2.14, 3.78), $p < 0.001$). Adverse impacts were also more likely for participants who were unemployed / not working (OR = 2.11 (1.31, 3.41), $p < 0.001$), or retired (OR = 1.49 (1.20, 1.85), $p < 0.001$), compared to those who were employed, and participants living in flats (OR = 1.36 (1.00, 1.84), $p = 0.05$) compared to those living in a detached/semi-detached house. Beneficial impacts ('better' *vs.* no change) on productivity at home were also more likely to be reported by participants ≤34 years of age (OR = 1.61 (1.1., 2.34), $p = 0.01$) compared to ≥65, and those living in a bungalow (OR = 1.97 (1.24, 3.14), $p < 0.001$) compared to detached/semi-detached.

Impacts of the hot weather on mood were also influenced by gender, age, employment status and housing type (Table 1). Females had an increased odds of reporting 'worse' mood (OR = 1.78 (1.41, 2.25), $p < 0.001$). Being ≤34 years of age was associated with increased odds of both 'worse' mood (OR = 3.36 (2.31, 4.89), $p < 0.001$) and 'better' mood (OR = 1.38 (1.05, 1.83), $p = 0.02$) compared to being ≥65. In addition, being unemployed / not working, compared to employed (OR = 2.58 (1.56, 4.28), $p < 0.001$), and living in a flat compared to a semi/detached house (OR = 1.77 (1.24, 2.51), $p < 0.01$) were associated with increased odds of 'worse' mood.

References:

Crawley, M.J., 2013. *The R Book*. John Wiley & Sons.

Field, A., Field, Z. and Miles, J., 2012. *Discovering statistics using R*. Sage.

Venables, W.N. and Ripley, B.D., 2002. *Modern Applied Statistics with S* . Fourth. New York: Springer. https://www.stats.ox.ac.uk/pub/MASS4/

Supplementary Table S6 | Summary of best fit multinomial logistic regression model outputs. Qualitative descriptions of impacts on aspects of daily life were collapsed to compare 'worse' impacts, ('slightly' and 'a lot' worse combined) and 'better' impacts, ('slightly' and 'a lot' better combined), to 'no change', which was set as the reference level.

**Aspect of daily life**	Variables	χ^2	*p*	levels		**Better** *vs.* No Change		**Worse** *vs.* No Change	
						Odds Ratio (95% CI)	*p*	Odds Ratio (95% CI)	*p*
**Sleep quality**	Intercept					0.07 (0.04, 0.11)	< 0.001	1.11 (0.92, 1.33)	0.27
	Gender	32.33	<0.001	Male	*reference level*				
$\chi^2 = 128.90$				Female		1.08 (0.68, 1.71)	0.75	1.62 (1.37, 1.92)	< 0.001
	Age	61.24	<0.001	≥ 65	*reference level*				
p <0.001				35 - 64		1.17 (0.62, 2.19)	0.63	1.39 (1.12, 1.73)	< 0.001
				≤ 34		1.91 (0.90, 4.07)	0.09	2.78 (2.12, 3.66)	< 0.001
	Employment status	9.28	0.05	Employed	*reference level*				
				Unemployed /not working		0.62 (0.08, 4.80)	0.65	2.03 (1.15, 3.58)	0.01
				Retired		0.90 (0.50, 1.64)	0.74	1.12 (0.92, 1.38)	0.26
	Housing type	*Not included in the minimal adequate model*							
	Residential area	*Not included in the minimal adequate model*							
**Productivity at home**	Intercept					0.23 (0.16, 0.33)	< 0.001	0.29 (0.22, 0.38)	< 0.001
	Gender	72.99	<0.001	Male	*reference level*				
$\chi^2 = 184.48$				Female		1.18 (0.94, 1.48)	0.16	2.12 (1.77, 2.53)	< 0.001
	Age	63.83	<0.001	≥ 65	*reference level*				
p <0.001				35 - 64		1.19 (0.89, 1.61)	0.25	1.38 (1.09, 1.73)	0.01
				≤ 34		1.61 (1.10, 2.34)	0.01	2.84 (2.14, 3.78)	< 0.001
	Employment status	24.48	<0.001	Employed	*reference level*				
				Unemployed /not working		0.83 (0.37, 1.85)	0.65	2.11 (1.31, 3.41)	< 0.001
				Retired		1.20 (0.90, 1.59)	0.21	1.49 (1.20, 1.85)	< 0.001
	Housing type	13.53	0.04	(Semi) Detached	*reference level*				
				Terrace		0.90 (0.68, 1.20)	0.48	0.98 (0.80, 1.20)	0.83
				Flat		1.00 (0.64, 1.55)	0.99	1.36 (1.00, 1.84)	0.05
				Bungalow		1.97 (1.24, 3.14)	< 0.001	1.31 (0.88, 1.96)	0.19
	Residential area	*Not included in the minimal adequate model*							

Supplementary Table S6 continued

**Aspect of daily life**	Variables	χ^2	*p*	levels		**Better** *vs.* No Change		**Worse** *vs.* No Change	
						Odds Ratio (95% CI)	*p*	Odds Ratio (95% CI)	*p*
	Intercept			-		0.70 (0.54, 0.90)	0.01	0.15 (0.10, 0.22)	< 0.001
**Mood**	Gender	25.16	<0.001	Male	*reference level*				
$\chi^2 = 174.16$				Female		1.11 (0.93, 1.31)	0.24	1.78 (1.41, 2.25)	< 0.001
p <0.001	Age	72.42	<0.001	≥ 65	*reference level*				
				35 - 64		1.15 (0.92, 1.43)	0.22	1.21 (0.88, 1.66)	0.25
				≤ 34		1.38 (1.05, 1.83)	0.02	3.36 (2.31, 4.89)	< 0.001
	Employment status	22.60	<0.001	Employed	*reference level*				
				Unemployed /not working		0.85 (0.50, 1.45)	0.55	2.58 (1.56, 4.28)	< 0.001
				Retired		1.15 (0.93, 1.41)	0.19	1.29 (0.96, 1.74)	0.09
	Housing type	16.71	0.01	(Semi) Detached	*reference level*				
				Terrace		0.97 (0.79, 1.19)	0.77	0.90 (0.69, 1.17)	0.42
				Flat		1.18 (0.85, 1.64)	0.33	1.77 (1.24, 2.51)	< 0.001
				Bungalow		1.40 (0.95, 2.06)	0.09	1.50 (0.92, 2.45)	0.11
	Residential area	*Not included in the minimal adequate model*							

Supplementary Table S7 | Changes to daily activities in response to heat-health warnings and extreme hot weather reported by 34% of survey participants in free-text responses. Where multiple actions were taken by a single participant, each action was weighted (1/number of actions).

Themes	Sub-themes	Actions	Sum of weighted actions	% actions taken
**Routine**	-	-	**422**	**39**
	**Alternative plans**	-	137	12
		More time indoors	70	6
		Worked from home	21	2
		Changed activities	20	2
		Transport	19	2
		Worked in office (air con)	7	1
	**Time of day**	-	116	11
		Dog walk / animal care	47	4
		Exercise	20	2
		General	18	2
		Work	15	1
		Chores	14	1
		Meals	3	<1
	**Reduced activity**	**-**	99	9
		Physical activity / exercise	43	4
		Less active / cancelled plans	42	4
		Stayed home	14	1
	**Benefit**	-	65	6
		Outside more	28	3
		Holiday	17	2
		Coast	13	1
		More physically active	6	1
	**Other**	-	5	<1
**Personal Care**	-	-	**283**	**26**
	**Physical health**	-	95	9
		Increased fluids	81	7
		Changed meals	10	1
		Siesta	4	<1
	**Cooling**	-	79	7
		Lighter clothing	34	3
		Cold drinks / ice cream	25	2
		Ice packs/ cold shower	12	1
		Paddling / swimming	8	1

Themes	Sub-themes	Actions	Sum of weighted actions	% actions taken
	**Sun Protection**	-	55	5
		Sought shade	20	2
		Sunscreen	14	1
		Avoided sun	14	1
		Hat / parasol	7	1
	**Other**	-	5	<1
**Technology Solutions**	-	-	**247**	**23**
	-	Fan	191	17
		Air conditioning	52	5
		Other	4	<1
**Home Adaptations**	-	-	**127**	**12**
	-	Closed curtains / blinds	72	7
		Lighter bedding	18	2
		Opened windows	16	1
		Closed windows	12	1
		Other	9	1
**Other**	-	-	**16**	**1**

Supplementary Table S8 | Changes to daily activities in response to heat-health warnings and extreme hot weather reported in free-text survey responses, broken down by participant characteristics.

		% survey participants undertaking behavioural adaptations grouped by themes					
		**No Action**	**Routine**	**Personal Care**	**Technology Solutions**	**Home Adaptations**	**Other**
**Overall**	**-**	**66**	**13**	**9**	**8**	**4**	**1**
Gender	Female	64	14	9	8	4	1
	Male	72	10	7	7	3	0
Age	≤ 34	66	12	9	11	2	1
	35 – 64	67	13	9	6	5	0
	≥ 65	64	15	9	6	5	0
Employment	Employed	68	11	9	8	3	0
	Unemployed or not working	67	15	7	7	3	2
	Retired	63	16	13	10	3	0
Housing Type	(Semi) Detached	66	13	9	7	4	1
	Terrace	69	12	8	7	3	0
	Flat	64	15	8	10	2	0
	Bungalow	60	16	10	7	7	0
Residential Area	Urban	67	14	8	8	2	0
	Suburbs	66	12	9	8	4	0
	Rural	66	15	8	6	4	1
